# Soy isoflavones avert chronic inflammation-induced bone loss and vascular disease

**DOI:** 10.1186/1476-9255-4-17

**Published:** 2007-09-07

**Authors:** Elizabeth A Droke, Kelly A Hager, Megan R Lerner, Stan A Lightfoot, Barbara J Stoecker, Daniel J Brackett, Brenda J Smith

**Affiliations:** 1 Department of Nutrition, Food Science and Hospitality, South Dakota State University, Brookings, SD 57006, USA; 2 Department of Nutritional Sciences, Oklahoma State University, Stillwater, OK 74078, USA; 3 Department of Surgery, University of Oklahoma Health Sciences Center, Oklahoma City, OK 73190, USA; 4 Veterans Affairs Medical Center, Oklahoma City, OK 73190, USA; 5 Department of Pathology, University of Oklahoma Health Sciences Center, Oklahoma City, OK 73190, USA; 6 Department of Medicine, University of Oklahoma Health Sciences Center, Oklahoma City, OK 73190, USA

## Abstract

**Background:**

Evidence from epidemiological, clinical and animal studies suggests a link may exist between low bone density and cardiovascular disease, with inflammatory mediators implicated in the pathophysiology of both conditions. This project examined whether supplementation with soy isoflavones (IF), shown to have anti-inflammatory properties, could prevent tissue expression of TNF-α and the development of skeletal pathology in an animal model of chronic inflammation.

**Methods:**

Eight-week old, intact, female C57BL/6J mice were used. In Phase 1, a lipopolysaccharide (LPS)-dose response study (0, 0.133, 1.33 and 13.3 μg/d) was conducted to determine the LPS dose to use in Phase 2. The results indicated the 1.33 μg LPS/d dose produced the greatest decrease in lymphocytes and increase in neutrophils. Subsequently, in Phase 2, mice were randomly assigned to one of six groups (n = 12–13 per group): 0 or 1.33 μg LPS/d (placebo or LPS) in combination with 0, 126 or 504 mg aglycone equivalents of soy IF/kg diet (Control, Low or High dose IF). Mice were fed IF beginning 2 wks prior to the 30-d LPS study period.

**Results:**

At the end of the study, no differences were detected in final body weights or uterine weights. In terms of trabecular bone microarchitecture, μCT analyses of the distal femur metaphysis indicated that LPS significantly decreased trabecular bone volume (BV/TV) and number (TbN), and increased separation (TbSp). Trabecular bone strength (i.e. total force) and stiffness were also compromised in response to LPS. The High IF dose provided protection against these detrimental effects on microarchitecture, but not biomechanical properties. No alterations in trabecular thickness (TbTh), or cortical bone parameters were observed in response to the LPS or IF. Immunohistomchemical staining showed that tumor necrosis factor (TNF)-α was up-regulated by LPS in the endothelium of small myocardial arteries and arterioles as well as the tibial metaphysis and down-regulated by IF.

**Conclusion:**

These results suggest IF may attenuate the negative effects of chronic inflammation on bone and cardiovascular health. Additional research is warranted to examine the anti-inflammatory properties of the soy isoflavones and the mechanisms underlying their prevention of chronic inflammation-induced bone loss.

## Background

Osteopenia is a common complication with conditions associated with chronic elevation of pro-inflammatory mediators and has been linked to increased incidence of cardiovascular morbidity and mortality [1,2]. This association between low bone density and vascular disease is supported by population studies [1-4] and clinical evidence [2,5,6], including the recent observation that cardiovascular disease risk in postmenopausal women increased relative to the severity of their osteopenia [7]. The relationship between the immune, skeletal and cardiovascular systems is further demonstrated in patients with autoimmune diseases such as rheumatoid arthritis [8,9] and lupus erythematosus who experience significant bone loss and increased risk of cardiovascular disease. In a recent review, Lessem [10] examined the association between atherosclerosis and alveolar bone loss and suggested the development of atherosclerotic plaques may be related to a long-term burden of infection. In a rodent model of chronic inflammation, we have recently demonstrated that a 90 day exposure to LPS results in a decrease in bone density localized to the trabecular bone, pervascular fibrosis and disruption of the intima in intramural arteries. This provides further evidence to support a link among the immune, skeletal and cardiovascular systems. Thus, the association between reduced bone mass and increased risk of cardiovascular disease may be due in part to the presence of a persistent inflammatory state.

The dysregulation of pro- versus anti-inflammatory mediators is characteristic of autoimmune diseases and chronic infections and has been implicated as a potential mechanism involved in the etiology of skeletal decalcification and cardiovascular diseases [11-14]. Increased tumor necrosis factor (TNF)-α expression has been reported in response to estrogen deficiency which coincides with increased bone loss and cardiovascular disease risk associated with menopause [15]. These imbalances in pro- and anti-inflammatory mediators and the failure to resolve the inflammatory response can have a significant impact on the health of an individual [16]. Therefore, intervention strategies targeting these inflammatory pathways may prevent inflammation-induced concomitant bone and vascular disease.

Epidemiological studies have demonstrated a reduced mortality rate due to coronary heart disease in populations consuming soy [17] and other evidence also suggests the isoflavones (IF) from soybeans may have anti-inflammatory activity in cardiovascular disease [18]. A number of studies have reported decreases in cytokines and inflammation with either soy foods or IF [19-21]; however, other research has not observed beneficial effects of soy isoflavones on markers of inflammation [22,23]. Genistein, the most abundant IF in soy, is a tyrosine kinase inhibitor [24] and thus may affect signaling pathways of immune cells and the subsequent innate and adaptive immune responses. The evidence related to the osteoprotective effects of soy IF on skeletal health has been somewhat equivocal [25,26]. Several animal studies have demonstrated soy IF prevention of bone loss due to ovarian hormone deficiency in rats [27-30], while others have not observed this same bone protective response in rats [26] as well as macaque monkeys [31]. Similarly, the data on soy IF and bone health from clinical trials have been variable, but promising. Supplementation with soy IF or soy containing foods have generally resulted in improved bone biomarkers [32,33], but had varying effects on bone density [32,34]. More recently, an association between soy consumption and fracture risk in early menopausal women was observed in the Shanghai Women's Health Study [35].

Based upon the evidence outlined above, soy IF provide a reasonable dietary intervention to consider in the prevention of chronic inflammation-induced bone loss and cardiovascular diseases. Though most of the soy IF studies have focused on either the skeletal or cardiovascular systems, the ability of soy IF to modulate the underlying inflammatory response involved in both of these conditions has not been assessed. Therefore, the purpose of this study was two-fold. First, to determine if increasing levels of soy IF prevents LPS-induced alterations in bone microarchitectural and biomechanical properties. Second, to determine if these effects of soy IF were associated with alterations in local (bone and heart) expression of the proinflammatory mediator TNF-α.

## Methods

### Animals

Eight-week old, intact, female C57BL/6J mice (Charles River Laboratories) were housed in an environmentally controlled animal care facility and allowed to acclimate for 1 wk prior to the start of each experiment. Mice were fed their respective diets and maintained on deionized water throughout the entire study period. At the termination of each study, animals were anesthetized and tail blood samples collected for the determination of differential leukocyte counts by manual microscopy from a peripheral blood smear. The mice were bled via the descending aorta and bone and heart specimens collected. Uteri were removed, cleaned of fat tissue and weighed to determine the presence of an estrogenic effect due to the soy IF. All animal procedures were conducted under an animal protocol approved by the Oklahoma State University Institutional Animal Care and Use Committee.

### Implantation of slow-release pellets

Lipopolysaccharide (E. coli Serotype 0127:B8; Sigma, St. Louis, MO) was incorporated into slow-release pellets designed to provide a consistent dose for 30 days (Innovative Research of America, Sarosota, FL) and implanted using the method of Smith et al [36]. In short, the pellets were subcutaneously implanted in the dorsal region of the neck, while the animals were anesthetized with isoflurane.

### Experimental design and treatments

This study was conducted in two phases: Phase 1 – a LPS dose-response study to determine the LPS dose to use in Phase 2; and, Phase 2 – the effects of soy IF in mice implanted with the slow-release LPS pellets to simulate chronic inflammation.

In Phase 1, 4 doses of LPS were used which were chosen based upon previous research in male Sprague Dawley rats [36]: 0, 0.133, 1.33, and 13.33 μg LPS/d. Mice (12 per group) were randomly assigned to treatment groups and fed a semi-purified diet (AIN-93G) throughout the entire study.

In Phase 2, a randomized control design with a factorial arrangement of treatments was used. After acclimation, mice were fed a semi-purified diet (AIN-93G) for two weeks and then weighed and randomly assigned to one of six treatments (n = 12 -13 per group): a placebo (pellet containing matrix only) or LPS (1.33 μg LPS/d) in combination with 0, 126 or 504 mg aglycone equivalents of soy IF/kg diet (0, 200 or 800 mg total IF/kg diet; designated as control, low or high respectively). The IF were provided as Prevastein 40 Isoflavone concentrate from Solae Company (St. Louis, MO) which contained 7.95% daidzein, 16.9% genistein and 0.36% glycitein or a total of 25.2% IF (expressed as aglycone equivalents). Mice were fed their respective treatment diets beginning 2 weeks prior to implantation with either a placebo or LPS pellet and then remained on their respective treatment diets for the 30-d LPS challenge period.

### Microcomputed tomography (μCT) analysis

The influence of chronic inflammation on trabecular and cortical bone microarchitecture was assessed at the femur distal metaphysis and middiaphysis. Each specimen was scanned using μCT (μCT40, SCANCO Medical, Switzerland) beginning at the distal growth plate in the proximal direction 300 slices (~12 μm/slice) for trabecular bone analyses followed by a scan of the midshaft region (i.e. ~38 slices) for assessment of cortical parameters [37]. An integration time of 70 milliseconds per projection was used for each scan with a rotational step of 0.36 degrees resulting in a total acquisition time of 150 minutes/sample. Trabecular bone was analyzed by placing contours beginning 25 slices from the growth plate to include only secondary spongiosa within the volume of interest (VOI). This region included 150 images using 1024 × 1024 matrix resulting in an isotropic voxel resolution of 22 μm3 and the following trabecular bone parameters were evaluated: bone volume (BV/TV), number (TbN), separation (TbSp), thickness (TbTh), structure model index (SMI), connectivity density (Conn Den) and linear attenuation (Lin Atten). Cortical analyses were performed by semi-automatically placing contours on 30 images in the midshaft region. Cortical thickness, cortical surface, medullary area, and porosity were determined. The operator conducting the scan analysis was blinded to the treatments associated with the specimen. Coefficients of variation (CVs) were 2.0% (BV/TV), 1.1% (TbN), 0.66% (TbTh) and 1.30% (TbSp) for morphometric and 4.6% (Conn Den) and 2.7% (SMI) for non-metric parameters.

### Biomechanical assessment using finite element analyses

Simulated compression testing was performed on the trabecular VOI generated from the μCT analyses to assess trabecular bone biomechanical strength at the distal femur metaphysis. Apparent mechanical properties chosen for each bone included: linear, elastic and isotropic with a Poisson's ratio of 0.3 and a Young's modulus of 10GPa [38]. Simulated compression testing was performed on the VOI from the scan of each distal metaphysis. The finite element (FE) software package (SCANCO Medical) was utilized for these analyses and physiological force, stiffness, size-independent stiffness, and von Mises stresses were determined.

### Immunohistochemical staining

At the time of necropsy, tibia and heart specimens were excised and immediately placed in 10% neutral-buffered formalin. To determine the alterations in TNF-α expression in the myocardium, corresponding vasculature, and bone, immunohistochemical (IHC) staining was performed. Longitudinal sections of decalcified tibia (7 μm) were cut from paraffin embedded specimens, mounted onto Superfrost/Plus slides (Fisher Scientific, Fair Lawn, NJ) and then rehydrated and washed 3X in PBS/Tween 20 (PBS/T; Sigma, St Louis MO). The bone sections were processed for immunohistochemistry using R&D Systems HRP-DAB goat kit. In short, sections were treated with peroxidase blocking reagent, washed with PBS/T and blocked with serum blocking reagent. After incubation with normal serum, sections were treated with Avidin/Biotin blocking reagents and placed in a humidified chamber overnight at 4°C with a 1:15 dilution of anti-mouse TNF-α/TNFSF1A antibody (R&D Systems). Sections were then washed, incubated with biotinylated secondary antibody, washed again and incubated with high sensitivity streptavidin conjugated to HRP (R&D Systems HRP-DAB goat kit). After rinsing with PBS/T slides were incubated with DAB chromogen for visualization and counterstained with Immuno * Master Hematoxylin (American Master * Tech Scientific, Inc., Lodi, CA). Controls were incubated with omission of the primary antibody.

The myocardial cross-sections of the heart (5 μm) were processed for immunohistochemistry using UltraVision LP Detection System HRP Polymer & AEC chromogen kit (Lab Vision Corporation, Fremont, CA). Sections were treated with DAKO^® ^Peroxidase Blocking Reagent (DAKO Corporation, Carpinteria, CA) to inhibit endogenous peroxidase activity followed by 4X PBS/T washes. Antigen retrieval was accomplished by placing slides in 10 mM citrate buffer, pH 6.0 in a steamer and cooled at room temperature. Tissue was blocked according to the manufacuturer's protocol for 5 minutes at room temperature and incubated with rabbit polyclonal antibody to TNF-α (dilution of 1:100; Abcam Inc, Cambridge, MA) at 4°C overnight. Sections were then washed with PBS/T 3X, incubated at room temperature with primary antibody enhancer, followed by 4 × washes in PBS/T and incubation with the HRP polymer. After rinsing with PBS/T, slides were incubated with AEC chromogen for visualization. Counterstaining was carried out with Immuno* Master Hematoxylin (American Master*Tech Scientific, Inc., Lodi, CA). Controls were incubated with omission of the primary antibody.

The amount of TNF-α expression in the bone and myocardial slides was scored by a pathologist who was blinded to treatments. The amount of cell involvement was determined using a scale of 0 to 4 with 0 representing no cells expressing staining; 1 representing 1–15% of cells expressing TNF-α; 2 representing 16–25% of cells expressing TNF-α; 3 representing 26–50% of cells expressing TNF-α; and, 4 representing > 50% of cells exhibiting TNF-α staining. The intensity of staining was also determined using a scale of 1 to 4 with 1 representing the least amount of staining and 4 representing the greatest amount of staining. The overall score was calculated by multiplying the categorical score for cell involvement by the categorical score for intensity of staining and was used in statistical analyses. The data for cell involvement and intensity of staining is expressed as the percent of animals receiving each score.

### Data presentation and statistical analysis

Statistical analysis was performed using PC SAS statistical software (version 8.02; SAS Institute Inc., Cary, NC). In Phase I the variables were analyzed by one-way ANOVA and in Phase II the variables were analyzed by two-way ANOVA with LPS and IF as factors. The ANOVA analyses were followed by post hoc analysis using the Fisher's least squares means separation test when F values were significant. Data are presented as means ± standard error (SE). For all analyses, a p < 0.05 was considered to be significantly different.

## Results

### Phase 1 differential counts

The percentage of lymphocytes was decreased (P < 0.05) (Placebo = 90.4 ± 2.3, Low = 85.2 ± 1.3, Medium = 75.5 ± 2.5, High = 65.7 ± 9.7) with increasing dose of LPS, while the percentage of neutrophils was increased (P < 0.05) from 4.5 ± 1.0 in the placebo group to 7.4 ± 1.9, 15.7 ± 1.5, and 12.0 ± 3.2 in the Low, Medium and High doses, respectively. Mice receiving the 1.33 μg LPS/d dose experienced the greatest change in the proportion of lymphocytes and neutrophils, while mice administered the highest dose did not show further changes. No differences (P > 0.05) were observed in the percentages of monocytes, eosinophils and basophils (data not shown). The three relatively low doses of LPS utilized in this study produced no detectable alterations in animal behavior in terms of grooming, food consumption and physical activity, but localized edema did develop around the pellet and was resolved within the first week of the study. Based upon the lymphocyte and neutrophil data, the medium dose of 1.33 μg LPS/d was chosen to induce the low grade inflammatory state to be used in Phase 2.

### Phase 2 body weights and uterine weights

As expected, no alterations (P > 0.05) in final body weights or uterine weights in response to soy IF or LPS were observed at the end of the study (Table 1). These data suggest soy IF, even at the high dose, did not have an estrogenic influence on the young, growing female mice. The absence of an effect on body weight combined with no noticeable alterations in food intake or grooming behavior, confirms our previous observation [36] in which chronic exposure to LPS at the dose used in the present study induced a low-grade inflammation but did not negatively impact basic animal behaviors.

**Table 1:** Body and uterine weight in mice fed soy isoflavones and administered LPS.

**Treatment**	**Body wt (g)**	**Uterine wt (g)**
**0 IF Placebo**	19.0 ± 0.3	0.12 ± 0.01
**0 IF LPS**	19.6 ± 0.4	0.11 ± 0.01
**126 IF Placebo**	19.6 ± 0.2	0.12 ± 0.01
**126 IF LPS**	19.4 ± 0.3	0.13 ± 0.01
**504 IF Placebo**	19.7 ± 0.3	0.11 ± 0.01
**504 IF LPS**	19.7 ± 0.2	0.13 ± 0.01
***P *value**		
**LPS**	0.4703	0.1457
**IF**	0.3844	0.4305
LPS*IF	0.3933	0.2276

Mice were fed soy isoflavones (IF; 0, 126 or 504 mg aglycone equivalents of IF/kg diet) for 14-days prior to and during a 30-day exposure to LPS (1.33 μg/d). Results are expressed as means ± standard error.

### Bone microarchitecture

Alterations in trabecular bone microarchitecture of the distal femur metaphysis and cortical parameters in the femur middiaphysis were analyzed using μCT. BV/TV (Table 2) was reduced (p < 0.05) by LPS in the 0 and low IF groups, but high IF protected (p < 0.05) against this detrimental effect on trabecular bone. As with BV/TV, chronic exposure to LPS decreased (p < 0.05) the TbN and increased (p < 0.05) TbSp in the 0 and low IF groups. The high IF protected (p < 0.05) against this deterioration in TbN and TbSp associated with chronic LPS administration (Table 2). TbTh and SMI (Table 2) were unaffected (p > 0.05) by either IF or LPS. Linear x-ray attenuation, indicative of trabecular bone density, tended (p = 0.06) to be reduced with high IF in the placebo group and enhanced with high IF under inflammatory conditions (Table 2). Trabecular bone connectivity density was reduced (p < 0.05) in the absence of LPS with high IF, but under inflammatory conditions this was not the case (Table 2). Cortical bone microarchitecture at the femur mid-diaphysis was also assessed. No alterations in cortical thickness, cortical area, medullary area or porosity were observed in conjunction with either LPS or IF at the end of the study period (Table 3).

**Table 2:** Alterations in microarchitectural properties of trabecular bone of the femur distal metaphysis.

**Treatment**	**BV/TV (%)**^A^	**TbN (1/mm**^3^**)**^B^	**TbSp (mm)**^C^	**TbTh (mm)**^D^	**Conn Den (1/mm**^3^**)**^E^	**SMI**^F^	**Linear Attenuation**
**0 IF Placebo**	8.80 ± 0.01^a^	3.89 ± 0.15^a, c^	0.27 ± 0.01^a^	0.051 ± 0.001	58.97 ± 8.14^a^	2.63 ± 0.10	1.01 ± 0.02
**0 IF LPS**	6.34 ± 0.01^b^	3.25 ± 0.15^b^	0.32 ± 0.01^b, d^	0.052 ± 0.001	40.56 ± 8.80^a, b^	2.83 ± 0.15	0.90 ± 0.06
**126 IF Placebo**	8.80 ± 0.01^a^	3.92 ± 0.10^a^	0.26 ± 0.01^a^	0.050 ± 0.002	47.33 ± 6.72^a, b^	2.91 ± 0.16	1.01 ± 0.02
**126 IF LPS**	6.52 ± 0.01^b^	3.41 ± 0.13^b, e^	0.30 ± 0.01^b, c, d^	0.050 ± 0.001	33.56 ± 3.80^b^	3.02 ± 0.05	0.95 ± 0.02
**504 IF Placebo**	7.14 ± 0.01^a, b^	3.50 ± 0.17^b, c, d^	0.29 ± 0.02^a, d^	0.050 ± 0.001	34.12 ± 4.99^b^	2.96 ± 0.14	0.96 ± 0.03
**504 IF LPS**	8.40 ± 0.01^a, b^	3.71 ± 0.14^a, d, e^	0.28 ± 0.01^a, c^	0.053 ± 0.002	52.12 ± 8.87^a, b^	2.93 ± 0.15	1.00 ± 0.02
***P *value**							
**LPS**	0.0731	0.0085	0.119	0.2834	0.4317	0.3779	0.1018
**IF**	0.9681	0.7839	0.5918	0.2478	0.4208	0.1493	0.6326
LPS*IF	0.0397	0.0099	0.0124	0.6706	0.0341	0.6773	0.0613

Mice were fed soy isoflavones (IF; 0, 126 or 504 mg aglycone equivalents of IF/kg diet) for 14-days prior to and during a 30-day exposure to LPS (1.33 μg/d). Results are expressed as means ± standard error. (A) trabecular bone volume (BV/TV), (B) trabecular number (TbN), (C) trabecular separation (TbSp), and (D) trabecular thickness (TbTh), (E) Connective Density (Conn Den), (F) Structure Model Index (SMI). Values for a given parameter that share the same superscript letter are not statistically different (P > 0.05) from each other.

**Table 3:** Alterations in microarchitectural properties of cortical bone of the femur mid-diaphysis.

**Treatment**	**Cortical Thickness (μm)**	**Cortical Area (mm**^2^**)**	**Medullary Area (mm**^2^**)**	**Porosity (%)**
**0 IF Placebo**	0.187 ± 0.003	0.625 ± 0.001	0.010 ± 0.001	0.98 ± 0.28
**0 IF LPS**	0.197 ± 0.003	0.646 ± 0.010	0.010 ± 0.002	0.84 ± 0.20
**126 IF Placebo**	0.193 ± 0.002	0.628 ± 0.014	0.009 ± 0.001	1.07 ± 0.22
**126 IF LPS**	0.191 ± 0.003	0.625 ± 0.008	0.012 ± 0.002	1.08 ± 0.31
**504 IF Placebo**	0.194 ± 0.005	0.625 ± 0.014	0.010 ± 0.001	0.99 ± 0.24
**504 IF LPS**	0.198 ± 0.001	0.652 ± 0.011	0.009 ± 0.001	0.86 ± 0.22
***P *value**				
**LPS**	0.1340	0.1086	0.4386	0.6895
**IF**	0.4189	0.5571	0.5553	0.7637
LPS*IF	0.1293	0.3899	0.1988	0.9444

Mice were fed soy isoflavones (IF; 0, 126 or 504 mg aglycone equivalents of IF/kg diet; 0, Low or High, respectively) for 14-days prior to and during a 30-day exposure to LPS (1.33 μg/d). Results are expressed as means ± standard error.

### Bone biomechanical properties

Data from simulated compression testing demonstrated the alterations in trabecular bone microarchitecture induced by the chronic inflammatory conditions had deleterious effects on bone biomechanical properties. Total and physiological compressive forces were significantly reduced by LPS and bone stiffness was also compromised (Table 4). Soy IF had no effect on these biomechanical properties, and even though the higher dose preserved many of the bone microarchitectural properties it was unable to protect against the detrimental effects of LPS on bone strength.

**Table 4:** Biomechanical properties of trabecular bone in the distal femur.

**Treatment**	**Total Force (N)**	**Physiological Force (N)**	**Stiffness (N/m × 10**^3^**)**	**Size Independent Stiffness (N/m**^2^**)**	**Von Mises Stress (MPa)**
**0 IF Placebo**	272.51 ± 33.62^a^	81.75 ± 10.09^a^	435.24 ± 52.46^a^	309.18 ± 36.02^a^	15.49 ± 1.41^a^
**0 IF LPS**	173.95 ± 56.94^b^	52.19 ± 17.08^b^	277.19 ± 90.74^b^	183.87 ± 58.53^b^	35.94 ± 10.22^b^
**126 IF Placebo**	272.43 ± 68.25^a^	81.73 ± 20.47^a^	432.74 ± 107.88^a^	299.34 ± 72.88^a^	25.75 ± 9.86^a^
**126 IF LPS**	111.29 ± 16.51^b^	33.39 ± 5.00^b^	177.58 ± 26.20^b^	128.93 ± 21.01^b^	32.19 ± 5.46^b^
**504 IF Placebo**	205.29 ± 40.16^a^	61.59 ± 12.05^a^	327.76 ± 64.41^a^	235.51 ± 46.35^a^	26.54 ± 8.11^a^
**504 IF LPS**	214.20 ± 65.75^b^	64.26 ± 17.73^b^	340.86 ± 104.08^b^	251.69 ± 78.52^b^	29.92 ± 7.83^b^
**P-values**					
**LPS**	0.0500	0.0500	0.0487	0.0500	0.1617
**IF**	0.8232	0.8232	0.8144	0.8148	0.8431
LPS*IF	0.2475	0.2475	0.2497	0.2388	0.9095

Mice were fed soy isoflavones (IF; 0, 126 or 504 mg aglycone equivalents of IF/kg diet) for 14-days prior to and during a 30-day exposure to LPS (1.33 μg/d). Biomechanical properties were determined using simulated compression strength testing with finite element analysis. Results are expressed as means ± standard error. Values for a given parameter that share the same superscript letter are not statistically different (P > 0.05) from each other.

### Histopathology

**Figure 1:**
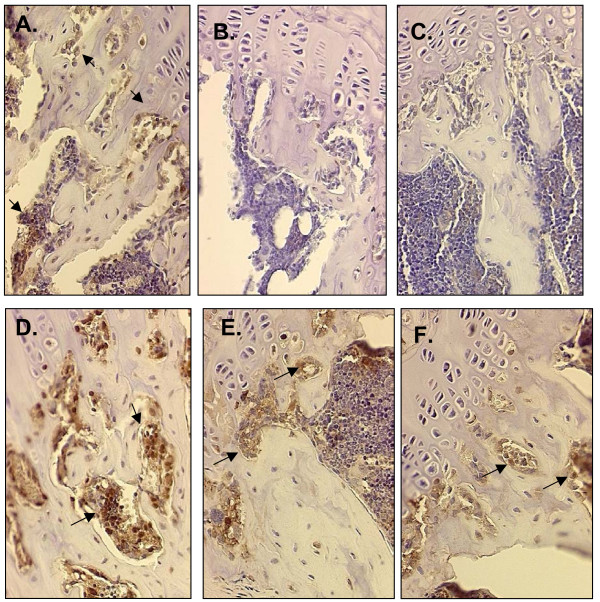
**Tumor-necrosis-α expression in proximal tibia metaphysis**. Micrographs (20x) from immunohistochemical staining for TNF-α in the proximal tibia metaphysis of mice following the feeding of soy isoflavones (IF; 0, 126 or 504 mg aglycone equivalents of IF/kg diet) for 14-days prior to and during a 30-day exposure to LPS (1.33 μg/d). Tibial sections shown are from mice administered: 0 LPS (placebo pellets) with either (A) 0 IF, (B) low IF (126 mg IF/kg diet), or (C) high IF (504 mg IF/kg diet); or LPS (pellets releasing 1.33 μg/d) with either (D) 0 IF, (E) low IF (126 mg IF/kg diet), or (F) high IF (504 mg IF/kg diet). The representative sections demonstrate an increase in expression of TNF-α with LPS (arrow in D) and a down-regulation of expression with the low (E) and high (F) IF doses.

To evaluate localized changes in proinflammatory cytokines implicated in the etiology of bone loss and cardiovascular diseases, the expression of TNF-α was evaluated in both bone and heart tissue. Up-regulation of TNF-α expression was evident in the metaphyseal region of the tibia after 30 days of exposure to LPS (Figure 1D). As can been seen in Figure 1E and 1F, both the low and high doses of IF down-regulated the LPS-induced TNF-α expression within this region of the bone. Table 5 shows the cell involvement and the intensity of staining in the bone tissue. As evidenced by the overall score for TNF-α expression, both the low and high doses of IF were able to prevent (P < 0.05) an increase in LPS-induced TNF-α expression.

**Table 5:** TNF-α expression in bone

**Score**		**Cells Expressing TNF-α**		**Intensity of TNF-α Expression**		Overall Score* (Cells Expressing × Intensity of Expression)
		**%**	X¯ MathType@MTEF@5@5@+=feaafiart1ev1aaatCvAUfKttLearuWrP9MDH5MBPbIqV92AaeXatLxBI9gBaebbnrfifHhDYfgasaacH8akY=wiFfYdH8Gipec8Eeeu0xXdbba9frFj0=OqFfea0dXdd9vqai=hGuQ8kuc9pgc9s8qqaq=dirpe0xb9q8qiLsFr0=vr0=vr0dc8meaabaqaciaacaGaaeqabaqabeGadaaakeaaieqacuWFybawgaqeaaaa@2E03@	**%**	X¯ MathType@MTEF@5@5@+=feaafiart1ev1aaatCvAUfKttLearuWrP9MDH5MBPbIqV92AaeXatLxBI9gBaebbnrfifHhDYfgasaacH8akY=wiFfYdH8Gipec8Eeeu0xXdbba9frFj0=OqFfea0dXdd9vqai=hGuQ8kuc9pgc9s8qqaq=dirpe0xb9q8qiLsFr0=vr0=vr0dc8meaabaqaciaacaGaaeqabaqabeGadaaakeaaieqacuWFybawgaqeaaaa@2E03@	
**0 IF Placebo**	1	0	2.0 ± 0.3	0	2.0 ± 0.0	5.0 ± 0.6^b^
	2	50		100		
	3	50		0		
	4	0		0		
**126 IF Placebo**	1	33.3	1.7 ± 0.3	100	1.0 ± 0.0	1.7 ± 0.3^c^
	2	66.6		0		
	3	0		0		
	4	0		0		
**504 IF Placebo**	1	33.3	1.7 ± 0.3	100	1.0 ± 0.0	1.7 ± 0.3^c^
	2	66.6		0		
	3	0		0		
	4	0		0		
**0 IF LPS**	1	0	4.0 ± 0.0	0	4.0 ± 0.0	16.0 ± 0.0^a^
	2	0		0		
	3	0		0		
	4	100		100		
**126 IF LPS**	1	33.3	1.3 ± 0.3	33.3	1.7 ± 0.3	2.0 ± 0.0^c^
	2	66.6		66.6		
	3	0		0		
	4	0		0		
**504 IF LPS**	1	100	1.0 ± 0.0	33.3	1.7 ± 0.3	1.7 ± 0.3^c^
	2	0		66.6		
	3	0		0		
	4	0		0		

Mice were fed soy isoflavones (IF; 0, 126 or 504 mg aglycone equivalents of IF/kg diet) for 14-days prior to and during a 30-day exposure to LPS (1.33 μg/d). TNF-α expression was determined immunhistochemically. Chi-square analysis was performed on the % of cells expressing TNF-α and the intensity of expression. The overall score was calculated. Results for the overall score are expressed as means ± standard error. Values for a given parameter that share the same superscript letter are not statistically different (P > 0.05) from each other. P-value for LPS*Diet on overall scores is < 0.0001.

**Figure 2:**
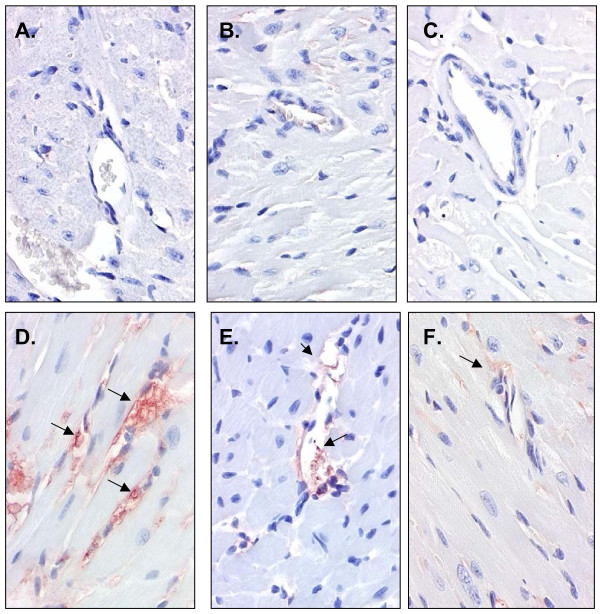
**Tumor-necrosis-α expression in myocardial tissue**. Representative cross-sections of the myocardium showing immunohistochemical staining for TNF-α in mice administered: 0 LPS (placebo pellets) with either (A) 0 IF, (B) low IF (126 mg IF/kg diet), or (C) high IF (504 mg IF/kg diet); or LPS (pellets releasing 1.33 μg/d) with either (D) 0 IF, (E) low IF (126 mg IF/kg diet), or (F) high IF (504 mg IF/kg diet). Micrographs (20x) show no endothelial expression of TNF-α in the placebo mice but a marked increase in the animals receiving LPS (note arrows indicating TNF-α expression). TNF-α expression was down-regulated with increasing dose of IF.

Similar results were observed in the myocardium (Figure 2). Chronic exposure (30-d) to LPS increased endothelial TNF-α expression in the small intramural arteries and arterioles (Figure 2D) which was down-regulated by the low and high doses of IF (Figure 2E &2F; Table 6).

**Table 6:** TNF-α expression in myocardial tissue.

**Score**		**Cells Expressing TNF**		**Intensity of TNF Expression**		Overall Score* (Cells Expressing × Intensity of Expression)
		**%**	X¯ MathType@MTEF@5@5@+=feaafiart1ev1aaatCvAUfKttLearuWrP9MDH5MBPbIqV92AaeXatLxBI9gBaebbnrfifHhDYfgasaacH8akY=wiFfYdH8Gipec8Eeeu0xXdbba9frFj0=OqFfea0dXdd9vqai=hGuQ8kuc9pgc9s8qqaq=dirpe0xb9q8qiLsFr0=vr0=vr0dc8meaabaqaciaacaGaaeqabaqabeGadaaakeaaieqacuWFybawgaqeaaaa@2E03@	**%**	X¯ MathType@MTEF@5@5@+=feaafiart1ev1aaatCvAUfKttLearuWrP9MDH5MBPbIqV92AaeXatLxBI9gBaebbnrfifHhDYfgasaacH8akY=wiFfYdH8Gipec8Eeeu0xXdbba9frFj0=OqFfea0dXdd9vqai=hGuQ8kuc9pgc9s8qqaq=dirpe0xb9q8qiLsFr0=vr0=vr0dc8meaabaqaciaacaGaaeqabaqabeGadaaakeaaieqacuWFybawgaqeaaaa@2E03@	
**0 IF Placebo**	1	0	0.00 ± 0.00	0	0.00 ± 0.00	0.00 ± 0.00^c^
	2	0		0		
	3	0		0		
	4	0		0		
**126 IF Placebo**	1	0	0.00 ± 0.00	0	0.00 ± 0.00	0.00 ± 0.00^c^
	2	0		0		
	3	0		0		
	4	0		0		
**504 IF Placebo**	1	0	0.00 ± 0.00	0	0.00 ± 0.00	0.00 ± 0.00^c^
	2	0		0		
	3	0		0		
	4	0		0		
**0 IF LPS**	1	12	2.88 ± 0.35	0	4.00 ± 0.00	11.50 ± 1.40^a^
	2	12		0		
	3	50		0		
	4	25		100		
**126 IF LPS**	1	33.3	1.67 ± 0.21	33.3	2.33 ± 0.56	4.33 ± 1.28^b^
	2	66.6		33.3		
	3	0		0		
	4	0		33.3		
**504 IF LPS**	1	71.4	2.00 ± 0.31	71.4	1.29 ± 0.18	2.86 ± 0.83^b^
	2	28.6		28.6		
	3	0		0		
	4	0		0		

Mice were fed soy isoflavones (IF; 0, 126 or 504 mg aglycone equivalents of IF/kg diet) for 14-days prior to and during a 30-day exposure to LPS (1.33 μg/d). TNF-α expression was determined immunhistochemically. Chi-square analysis was performed on the % of cells expressing TNF-α and the intensity of expression. The overall score was calculated. Results for the overall score are expressed as means ± standard error. Values for a given parameter that share the same superscript letter are not statistically different (P > 0.05) from each other. P-value for LPS*Diet on overall scores is < 0.0001.

## Discussion

Administration of LPS has been used extensively in *in vitro and in vivo *studies to evaluate the influence of the innate immune response on the skeletal and cardiovascular systems. Much of the research has used either a single injection of LPS to simulate an acute response [39] or repeated injections [40,41] or infusion [42] to simulate chronic inflammation; however, few of these models have been maintained for more than 2 weeks. Recently, Smith et al [36] utilized a slow-release pellet system impregnated with very low doses of LPS to study the influence of an inflammatory state over 90 days on bone metabolism, myocardial and vascular pathology in male Sprague Dawley rats. Continuous administration of LPS produced a persistent systemic inflammatory state characterized by up-regulation of proinflammatory molecules in bone and the vascular endothelium of the heart with concurrent trabecular bone loss. This same technique of administering LPS was used in the present study over 30 days in female mice in which we also observed up-regulation of the proinflammatory cytokine, TNF-α in bone and vascular tissue at 30 days. This further supports the presence of an ongoing chronic inflammatory state without alterations in animal behavior, suggestive of a very low grade inflammatory response.

In the present study, the LPS-induced inflammation in mice resulted in reduced trabecular bone characterized by a decrease in the number of trabeculae and an increase in the inter-trabecular space with subsequent decreases in bone biomechanical properties. An increase in TNF-α expression in trabecular bone was also observed with LPS administration suggesting a role for this cytokine in bone loss. Tumor necrosis factor-α stimulates osteoclast differentiation and activity resulting in an increase in bone resorption [43] and inhibits bone formation by decreasing osteoblast progenitor cell recruitment and increasing osteoblast apoptosis [40,41]. The alterations in bone combined with the myocardial and vascular changes induced by low grade inflammation provide further support for the theory that inflammatory mediators provide the pathophysiological link between these two disease processes. Interventions targeting changes in local proinflammatory cytokine expression and circulating leukocytes are thus warranted [39,44] in order to prevent the detrimental effects of chronic inflammation.

A variety of *in vitro*, *in vivo *and clinical studies have suggested that dietary soy isoflavones have anti-inflammatory properties thus potentially influencing inflammation-induced bone loss and vascular changes. The effects of soy IF on circulating proinflammatory cytokines, such as TNF-α, have been inconsistent which is likely due to differences in the amount and type of soy product consumed and the length of the study [45-47]. The discrepancies in the clinical findings may suggest the existence of a threshold IF concentration which is needed before beneficial effects will be observed. The beneficial effects of soy IF on proinflammatory cytokines may also be most relevant when mediated at the tissue level. In the present study, the LPS-induced TNF-α expression in vascular and bone tissue was down-regulated with IF. These observations suggest the IF may aid in the resolution of inflammation [48] at the tissue level thus averting the detrimental consequences of inflammation-induced bone loss and vascular changes that can lead to cardiovascular diseases.

Numerous pre-clinical and clinical studies have evaluated the ability of soy and its IF to retard or reverse bone loss with varied results [25,26,49]. In the present study, the high dose of IF (504 mg IF/kg diet) was able to protect against the deterioration of trabecular bone microarchitectural properties observed with chronic LPS administration. These effects may have been mediated to some degree by soy IF's ability to protect against the TNF-α-induced increase in osteoclast differentiation and activity, inhibition of osteoblast activity or perhaps both [50]. Furthermore, soy IF may also inhibit TNF-α-induced apoptosis in osteoblasts [51]. Despite the improvement in trabecular bone, it should be noted that in the present study soy IF were not able to completely protect against the harmful effects of inflammation on trabecular bone biomechanical properties as demonstrated by the simulated compression testing using finite element analysis. Although these data represent dissociation between trabecular microarchitecture and bone strength, it is unclear as to whether some intrinsic tissue quality was altered by chronic inflammation that could not be prevented by soy IF or if the short duration of the study was a determining factor. It should also be noted that the higher dose of IF (504 mg IF/kg diet) tended to reduce the trabecular bone microarchitectural properties of connective density and linear attenuation suggesting a detrimental effect of the higher IF concentrations on trabecular bone. These observations warrant further investigation due to the prevalent usage of IF supplements.

Our results suggest that by down-regulating inflammatory mediators such as TNF-α at the tissue level, soy IF may reduce the risk of cardiovascular diseases associated with chronic inflammation. However, it should be mentioned that the results of clinical trials have been mixed at best and it is unclear whether the benefits associated with soy are due to its isoflavones, fat, fiber or micronutrient content [52]. Whether soy IF's ability to reduce systemic levels of pro-inflammatory cytokines [45] or localized tissue expression as evidenced in the present study translates into cardiovascular disease risk reduction remains to be determined. Further studies are warranted to clarify whether it is the effects of soy IF on TNF-α alone or other inflammatory pathways that mediate these protective effects on bone and cardiovascular tissue.

Conclusion

Administration of LPS over 30 days using a slow-release pellet system produced a low grade chronic inflammation in mice that resulted in bone pathology and increased tissue expression of TNF-α. Dietary supplementation with soy IF was able to avert the detrimental effects of chronic inflammation on the skeletal system. This protection provided by soy IF occurred in conjunction with down-regulating TNF-α expression in both bone and vascular tissue suggesting that TNF-α may serve as a key link between the concomitant bone loss and development of cardiovascular disease in conditions of chronic inflammation. Further research is needed to delineate the mechanism(s) underlying the effects of soy IF on TNF-α as well as other inflammatory mediators.

## Abbreviations

ANOVA – analysis of variance

BV/TV – trabecular bone volume

IF – isoflavones

LPS – lipopolysaccharide

TbN – trabecular number

TbSp – trabecular spacing

TbTh – trabecular thickness

TNF-α – tumor necrosis factor-α

## Competing interests

The author(s) declare that they have no competing interests.

## Authors' contributions

ED and BSmith contributed to the development of the project idea, study design and coordination, sample collection, and the drafting, editing and revising of the final manuscript. ED performed data analyses on the differential counts, body and uterine weights. BSmith contributed to analyses of bone parameters and performed data analyses on the bone parameters. KH was a graduate student on the project, assisted with day-to-day care of animals and sample collection, and contributed to the analyses of bone microarchitecture. ML performed the immunohistochemcial preparations of heart and bone tissue. SL was the pathologist who did the immunohistochemical scoring of the heart and bone tissue. BStoecker contributed to the analyses of bone microarchitecture. DB contributed contributed to the analyses of the heart and bone tissue. All authors have read and approved the final manuscript.

## References

[B1] von der Recke P, Hansen MA, Hassager C (1999). The association between low bone mass at the menopause and cardiovascular mortality. Am J Med.

[B2] Kado DM, Browner WS, Blackwell T, Gore R, Cummings SR (2000). Rate of bone loss is associated with mortality in older women: a prospective study. J Bone Miner Res.

[B3] Hak AE, Pols HA, van Hemert AM, Hofman A, Witteman JC (2000). Progression of aortic calcification is associated with metacarpal bone loss during menopause: a population-based longitudinal study. Arterioscler Thromb Vasc Biol.

[B4] Kiel DP, Kauppila LI, Cupples LA, Hannan MT, O'Donnell CJ, Wilson PW (2001). Bone loss and the progression of abdominal aortic calcification over a 25 year period: the Framingham Heart Study. Calcif Tissue Int.

[B5] van der Klift M, Pols HA, Hak AE, Witteman JC, Hofman A, de Laet CE (2002). Bone mineral density and the risk of peripheral arterial disease: the Rotterdam Study. Calcif Tissue Int.

[B6] Kenny AM, Boxer R, Walsh S, Hager WD, Raisz LG (2006). Femoral bone mineral density in patients with heart failure. Osteoporos Int.

[B7] Tanko LB, Christiansen C, Cox DA, Geiger MJ, McNabb MA, Cummings SR (2005). Relationship between osteoporosis and cardiovascular disease in postmenopausal women. J Bone Miner Res.

[B8] Romas E, Gillespie MT, Martin TJ (2002). Involvement of receptor activator of NFkappaB ligand and tumor necrosis factor-alpha in bone destruction in rheumatoid arthritis. Bone.

[B9] Mikuls TR (2003). Co-morbidity in rheumatoid arthritis. Best Pract Res Clin Rheumatol.

[B10] Lessem J (2005). Periodontitis in cardiology--a clinical perspective. J Int Acad Periodontol.

[B11] Loos BG (2005). Systemic markers of inflammation in periodontitis. J Periodontol.

[B12] McFarlane SI, Muniyappa R, Shin JJ, Bahtiyar G, Sowers JR (2004). Osteoporosis and cardiovascular disease: brittle bones and boned arteries, is there a link?. Endocrine.

[B13] Ross R (1999). Atherosclerosis--an inflammatory disease. N Engl J Med.

[B14] Willerson JT, Ridker PM (2004). Inflammation as a cardiovascular risk factor. Circulation.

[B15] Baldini V, Mastropasqua M, Francucci CM, D'Erasmo E (2005). Cardiovascular disease and osteoporosis. J Endocrinol Invest.

[B16] Elenkov IJ, Iezzoni DG, Daly A, Harris AG, Chrousos GP (2005). Cytokine dysregulation, inflammation and well-being. Neuroimmunomodulation.

[B17] Kagan A, Harris BR, Winkelstein W, Johnson KG, Kato H, Syme SL, Rhoads GG, Gay ML, Nichaman MZ, Hamilton HB, Tillotson J (1974). Epidemiologic studies of coronary heart disease and stroke in Japanese men living in Japan, Hawaii and California: demographic, physical, dietary and biochemical characteristics. J Chronic Dis.

[B18] Cassidy A, Hooper L (2006). Phytoestrogens and cardiovascular disease. J Br Menopause Soc.

[B19] Hall WL, Vafeiadou K, Hallund J, Bugel S, Koebnick C, Reimann M, Ferrari M, Branca F, Talbot D, Dadd T, Nilsson M, Dahlman-Wright K, Gustafsson JA, Minihane AM, Williams CM (2005). Soy-isoflavone-enriched foods and inflammatory biomarkers of cardiovascular disease risk in postmenopausal women: interactions with genotype and equol production. Am J Clin Nutr.

[B20] Curran EM, Judy BM, Newton LG, Lubahn DB, Rottinghaus GE, Macdonald RS, Franklin C, Estes DM (2004). Dietary soy phytoestrogens and ERalpha signalling modulate interferon gamma production in response to bacterial infection. Clin Exp Immunol.

[B21] Verdrengh M, Jonsson IM, Holmdahl R, Tarkowski A (2003). Genistein as an anti-inflammatory agent. Inflamm Res.

[B22] Blum A, Lang N, Peleg A, Vigder F, Israeli P, Gumanovsky M, Lupovitz S, Elgazi A, Ben-Ami M (2003). Effects of oral soy protein on markers of inflammation in postmenopausal women with mild hypercholesterolemia. Am Heart J.

[B23] Hilpert KF, Kris-Etherton PM, West SG (2005). Lipid response to a low-fat diet with or without soy is modified by C-reactive protein status in moderately hypercholesterolemic adults. J Nutr.

[B24] Akiyama T, Ishida J, Nakagawa S, Ogawara H, Watanabe S, Itoh N, Shibuya M, Fukami Y (1987). Genistein, a specific inhibitor of tyrosine-specific protein kinases. J Biol Chem.

[B25] Setchell KDR, Lydeking-Olsen E (2003). Dietary phytoestrogens and their effect on bone: evidence from in vitro and in vivo, human observational, and dietary intervention studies. American Journal of Clinical Nutrition.

[B26] Weaver CM, Cheong JM (2005). Soy isoflavones and bone health: the relationship is still unclear. J Nutr.

[B27] Arjmandi BH, Alekel L, Hollis BW, Amin D, Stacewicz-Sapuntzakis M, Guo P, Kukreja SC (1996). Dietary soybean protein prevents bone loss in an ovariectomized rat model of osteoporosis. J Nutr.

[B28] Arjmandi BH, Birnbaum R, Goyal NV, Getlinger MJ, Juma S, Alekel L, Hasler CM, Drum ML, Hollis BW, Kukreja SC (1998). Bone-sparing effect of soy protein in ovarian hormone-deficient rats is related to its isoflavone content. Am J Clin Nutr.

[B29] Fanti P, Monier-Faugere MC, Geng Z, Schmidt J, Morris PE, Cohen D, Malluche HH (1998). The phytoestrogen genistein reduces bone loss in short-term ovariectomized rats. Osteoporos Int.

[B30] Picherit C, Coxam V, netau-Pelissero C, Kati-Coulibaly S, Davicco MJ, Lebecque P, Barlet JP (2000). Daidzein is more efficient than genistein in preventing ovariectomy-induced bone loss in rats. J Nutr.

[B31] Register TC, Jayo MJ, Anthony MS (2003). Soy phytoestrogens do not prevent bone loss in postmenopausal monkeys. J Clin Endocrinol Metab.

[B32] Arjmandi BH, Lucas EA, Khalil DA, Devareddy L, Smith BJ, McDonald J, Arquitt AB, Payton ME, Mason C (2005). One year soy protein supplementation has positive effects on bone formation markers but not bone density in postmenopausal women. Nutr J.

[B33] Yamori Y, Moriguchi EH, Teramoto T, Miura A, Fukui Y, Honda KI, Fukui M, Nara Y, Taira K, Moriguchi Y (2002). Soybean isoflavones reduce postmenopausal bone resorption in female Japanese immigrants in Brazil: a ten-week study. J Am Coll Nutr.

[B34] Chen YM, Ho SC, Lam SS, Ho SS, Woo JL (2003). Soy isoflavones have a favorable effect on bone loss in Chinese postmenopausal women with lower bone mass: a double-blind, randomized, controlled trial. J Clin Endocrinol Metab.

[B35] Zhang X, Shu XO, Li H, Yang G, Li Q, Gao YT, Zheng W (2005). Prospective cohort study of soy food consumption and risk of bone fracture among postmenopausal women. Arch Intern Med.

[B36] Smith BJ, Lerner MR, Bu SY, Lucas  EA, Hanas JS, Lightfoot SA, Postier MS, Bronze RG, Brackett DJ (2006). Systemic bone loss and induction of coronary vessel disease in a rat model of chronic inflammation. Bone.

[B37] Muller R, Ruegsegger P (1996). Analysis of mechanical properties of cancellous bone under conditions of simulated bone atrophy.. J Biomech.

[B38] Newitt DC, Majumdar S, van RB, von IG, Harris ST, Genant HK, Chesnut C, Garnero P, MacDonald B (2002). In vivo assessment of architecture and micro-finite element analysis derived indices of mechanical properties of trabecular bone in the radius. Osteoporos Int.

[B39] Kadokami T, McTiernan CF, Kubota T, Frye CS, Bounoutas GS, Robbins PD, Watkins SC, Feldman AM (2001). Effects of soluble TNF receptor treatment on lipopolysaccharide-induced myocardial cytokine expression. Am J Physiol Heart Circ Physiol.

[B40] Armour KJ, Armour KE, van't Hof RJ, Reid DM, Wei XQ, Liew FY, Ralston SH (2001). Activation of the inducible nitric oxide synthase pathway contributes to inflammation-induced osteoporosis by suppressing bone formation and causing osteoblast apoptosis. Arthritis Rheum.

[B41] Dumitrescu AL, bd-El-Aleem S, Morales-Aza B, Donaldson LF (2004). A model of periodontitis in the rat: effect of lipopolysaccharide on bone resorption, osteoclast activity, and local peptidergic innervation. J Clin Periodontol.

[B42] Jarvelainen HA, Fang C, Ingelman-Sundberg M, Lindros KO (1999). Effect of chronic coadministration of endotoxin and ethanol on rat liver pathology and proinflammatory and anti-inflammatory cytokines. Hepatology.

[B43] Balga R, Wetterwald A, Portenier J, Dolder S, Mueller C, Hofstetter W (2006). Tumor necrosis factor-alpha: alternative role as an inhibitor of osteoclast formation in vitro. Bone.

[B44] Gabay C (2006). Interleukin-6 and chronic inflammation. Arthritis Res Ther.

[B45] Huang Y, Cao S, Nagamani M, Anderson KE, Grady JJ, Lu LJ (2005). Decreased circulating levels of tumor necrosis factor-alpha in postmenopausal women during consumption of soy-containing isoflavones. J Clin Endocrinol Metab.

[B46] Ryan-Borchers TA, Park JS, Chew BP, McGuire MK, Fournier LR, Beerman KA (2006). Soy isoflavones modulate immune function in healthy postmenopausal women. Am J Clin Nutr.

[B47] Jenkins DJ, Kendall CW, Connelly PW, Jackson CJ, Parker T, Faulkner D, Vidgen E (2002). Effects of high- and low-isoflavone (phytoestrogen) soy foods on inflammatory biomarkers and proinflammatory cytokines in middle-aged men and women. Metabolism.

[B48] Kadl A, Leitinger N (2005). The role of endothelial cells in the resolution of acute inflammation. Antioxid Redox Signal.

[B49] Reinwald S, Weaver CM (2006). Soy isoflavones and bone health: a double-edged sword?. J Nat Prod.

[B50] Nanes MS (2003). Tumor necrosis factor-alpha: molecular and cellular mechanisms in skeletal pathology. Gene.

[B51] Thammasitboon K, Goldring SR, Boch JA (2006). Role of macrophages in LPS-induced osteoblast and PDL cell apoptosis. Bone.

[B52] Sacks FM, Lichtenstein A, Van HL, Harris W, Kris-Etherton P, Winston M (2006). Soy protein, isoflavones, and cardiovascular health: an American Heart Association Science Advisory for professionals from the Nutrition Committee. Circulation.

